# Surgical management of intercondylar fractures of the humerus using triceps reflecting anconeus pedicle (TRAP) approach

**DOI:** 10.4103/0019-5413.33686

**Published:** 2007

**Authors:** Amite Pankaj, G Mallinath, Rajesh Malhotra, Surya Bhan

**Affiliations:** Department of Orthopedics, All India Institute of Medical Sciences, Ansari Nagar, New Delhi - 110 029, India

**Keywords:** Humerus, intercondylar fracture, Triceps-reflecting anconeus pedicle approach

## Abstract

**Background:**

Operative fixation of intra-articular fractures of the distal humerus requires adequate exposure. The transolecranon approach is a commonly used approach. The olecranon osteotomy has potential complications related to prominence/migration of hardware, displacement/nonunion of osteotomy and triceps weakness. Triceps-reflecting anconeus pedicle (TRAP) approach avoids the olecranon osteotomy without compromising the operative exposure. We present outcome of fixation of displaced intra-articular distal humeral fractures with the use of TRAP approach.

**Materials and Methods:**

We reviewed the functional and radiological results of 40 consecutive patients with intercondylar fractures of the humerus treated by internal fixation through TRAP approach. There were 28 males and 12 females and the average age was 32 ± 4.5 years. The right elbow was involved in 27 patients and the left elbow in 13 patients. The mechanism of injury was a fall in 20 patients, a motor-vehicle accident in 16 patients and direct trauma in four patients.

**Results:**

At a minimum follow-up of 12 months (average 18 ± 4 months) 35 (87.5%) patients had good triceps strength. The average range of motion was 118.4 ± 7 degrees (range 80°-130°). The average time to union was 3.2 ± 1.6 months (range two to six months). No patient had triceps rupture, implant failure, neurovascular deficit or nonunion. Two patients needed removal of the implant because of subcutaneous prominence.

**Conclusions:**

The TRAP approach provides good visualization for fixation of intercondylar fractures of the humerus, without any noticeable untoward effect on triceps strength and postoperative rehabilitation; and one can avoid iatrogenic fracture of the olecranon and its associated complications.

Distal humerus fractures demand technically difficult operative treatment, often with relatively high morbidity.^1^ The preferred treatment for displaced, intra-articular, intercondylar fractures of the distal part of the humerus is open reduction and internal fixation.^2^ Adequate exposure of the articular surface of the distal humerus and elbow joint is required for operative stabilization of bicolumnar distal humerus fractures. The transolecranon approach, which provides complete posterior visualization and access to the distal humerus, is the most commonly used surgical approach.^3^ An olecranon osteotomy and other extensor mechanism-disrupting approaches have risks and possible complications such as prominence/migration of hardware, displacement/nonunion of osteotomy and triceps weakness.

Need for better surgical visualization of fracture geometry has produced numerous new approaches and their modification. Surgical approaches to the elbow joint that dissociate the triceps from the olecranon have distinct disadvantages of more extensive dissection, triceps weakness/failure and delayed postoperative rehabilitation. Concerns regarding the potential complications of olecranon osteotomy have stimulated some authors to recommend triceps-splitting approach^4^ or triceps-reflecting approach.^3^

For these fracture patterns, an alternative exposure is the extensor mechanism-sparing paratricipital posterior approach to the distal humerus through a midline posterior incision, as suggested by O' Driscoll *et al*.^5^ This approach avoids an osteotomy and mobilizes the triceps and anconeus muscle off the posterior humerus and the intermuscular septae and provides adequate exposure for open reduction and internal fixation. Furthermore, this approach preserves neurovascular supply of anconeus, which is a dynamic stabilizer of the elbow.^5^ The purpose of our study was to determine the functional outcome of fixation of displaced intra-articular distal humeral fractures with use of triceps-reflecting anconeus pedicle approach.

## MATERIALS AND METHODS

Between July 2003 and June 2005, 60 patients with intercondylar fractures of the humerus were operated through triceps-reflecting anconeus pedicle (TRAP) approach. Patients with an ipsilateral or contralateral upper extremity injury or a preexisting musculoskeletal condition were excluded. Only patients who underwent plate fixation for both the medial and the lateral column through a posterior approach were included. Forty patients who had a minimum follow-up of 12 months are reported.

There were 28 males and 12 females. Average age was 32 years (range, 16-58 years). The right elbow was involved in 27 patients and left elbow in 13 patients. The mechanism of injury was a fall in 20 patients, a motor-vehicle accident in 16 patients and direct trauma in four patients. Thirty-five patients came to casualty within 12h and five patients reported one week after injury. Five patients had ulnar deficit at presentation. Only patients with T-condylar or intercondylar fractures, AO type C [17 C1, 15 C2 and 8 C3]^2^ were included for study. Grade I open wound was seen in four patients with widely displaced fractures.

### Surgical technique

**Figure 1:**
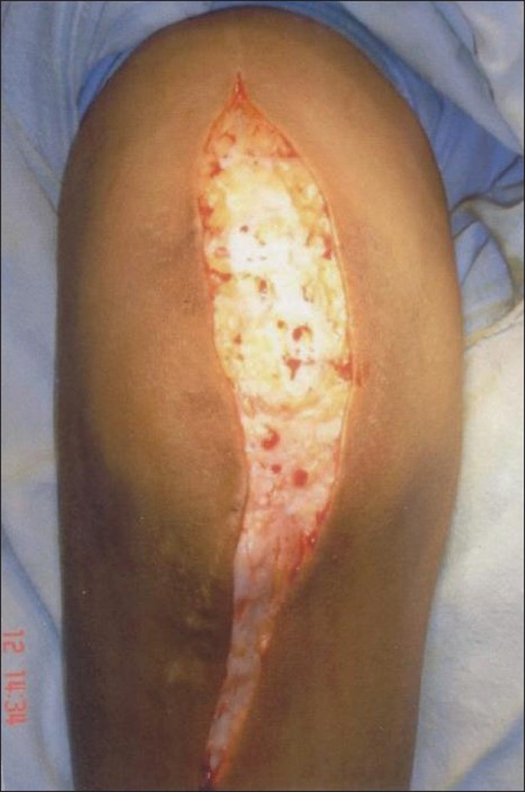
Skin incision for TRAP approach

All operations were done under general anesthesia. The patients were placed in lateral position and sterile tourniquet applied. We used the TRAP approach for exposure of the elbow as described by O' Driscoll *et al*.^5^ A straight posterior incision was made just lateral to the olecranon tip, approximately 10 cm proximal and 8 cm distal [Figure 1].

Medial and lateral skin flaps were raised to expose the supracondylar ridges on either side of the distal humerus. The ulnar nerve was first localized proximally where it emerged beneath the triceps tendon. The distal aspect of the intermuscular septum was released to increase the mobility of the ulnar nerve. The nerve was followed for at least 7 cm after the flexor pronator mass was entered. Its branches to the flexor carpi ulnaris were carefully preserved.

**Figure 2:**
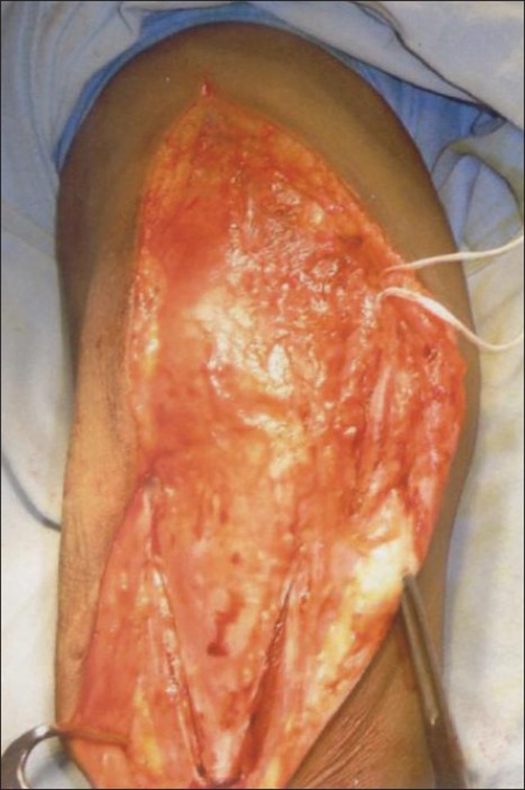
TRAP approach incorporates modified Kocker's approach on lateral side and a triceps reflecting approach on the medial side. Both approaches converge distally at the tip of the anconeus

Laterally, the flap was elevated to expose the interval between the anconeus and the extensor carpi ulnaris [Figure 2].

**Figure 3:**
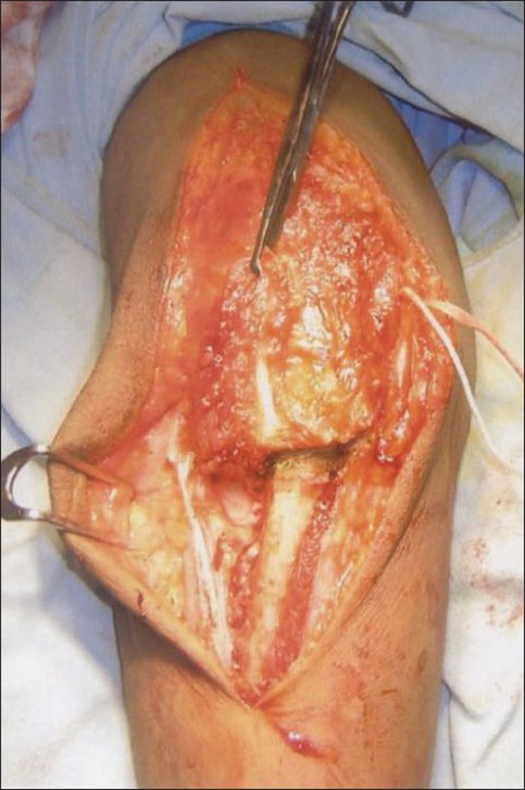
The anconeus-triceps flap is detached from its distal attachment and the flap is reflected to expose the lower end of the humerus

The anconeus-triceps flap was detached from its distal attachment (5-7 cm from the tip of olecranon) and dissected off the lateral side of the elbow and proximal ulna, preserving the integrity of the lateral collateral ligament complex, including annular ligament [Figure 3].

This was accomplished easily by commencing the dissection distally and working proximally. The posterior capsule was incised and the dissection was carried out proximally between the triceps and posterior humerus. The fibers of the deep head of the triceps were dissected off the posterior humerus by sharp and blunt dissection.

**Figure 4:**
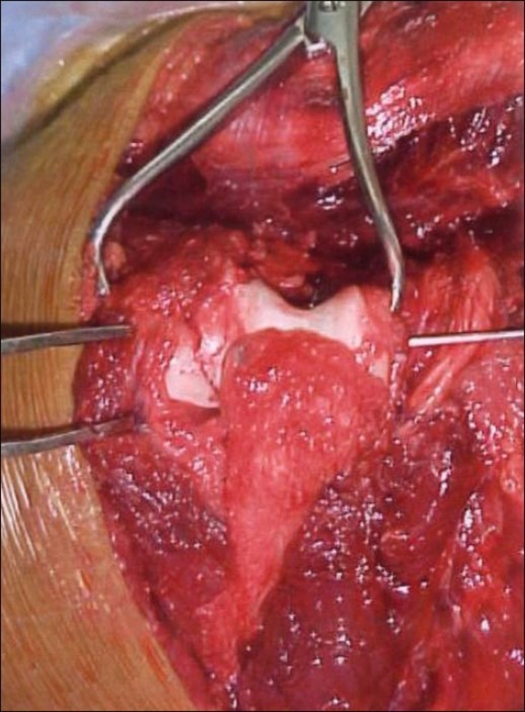
Good visualization of the fracture and an intact olecranon helps in reconstituting the distal humerus

**Figure 5:**
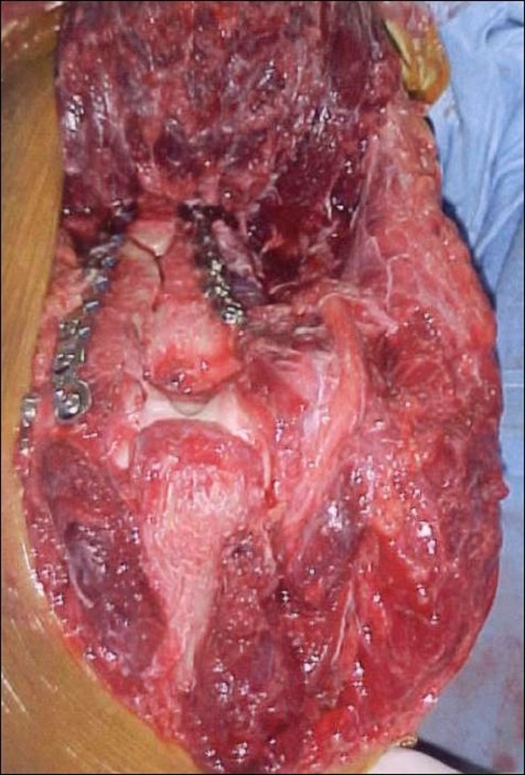
Both columns stabilized with 3.5 mm reconstruction plates applied on the dorsal surface

The intra-articular component was reduced first, after which the reconstituted condylar block was reduced and fixed provisionally to the medial and lateral columns with 1.6 or 2.0-mm Kirschner wires [Figure 4]. 3.5-mm reconstruction plates were contoured to fit along the involved columns. Both columns were plated along the posterior surface [Figure 5]. Corticocancellous bone graft from the iliac crest was used to fill the bone gaps in 10 patients.

Long screws aiming from the medial or lateral epicondyles through the medial and lateral columns provided additional fixation. Care was taken to ensure that the reconstructed humeral condyles did not narrow the trochlea and an intact olecranon process helped in reconstructing the articular surface. Ulnar nerve was transposed anteriorly in patients with preoperative symptoms of nerve involvement or if the hardware protruded medially in the course of nerve.

**Figure 6:**
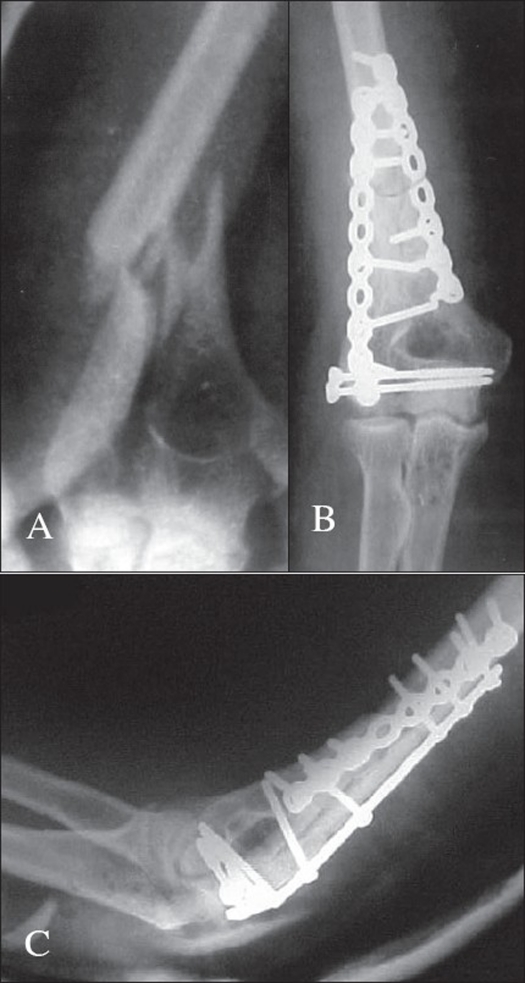
X-ray of elbow A) AP view of pre-operative showing fracture and B) AP view C) Lateral view of post-operative showing fracture union. The olecranon osteotomy is conspicuous by its absence

After intraoperative radiographs were made to confirm adequate placement of the hardware and reconstitution of the osseous anatomy, the elbow was moved through a range of motion to test the stability of the repair and also to guide the goals of postoperative rehabilitation. The triceps was reattached with interrupted number-2 braided polyester sutures, with use of drill-holes through bone in the region of the olecranon [Figure 6]. A hemovac drain was placed on the triceps fascia and the subcutaneous tissue and skin closed in layers. Above elbow POP slab was applied in 90° flexion for two weeks.

Gentle active and active-assisted range-of-motion exercises were started under the supervision of a physical therapist after two weeks. Active extension was prohibited until eight weeks postoperatively to avoid undue stress on the extensor mechanism repair.

### Assessment

Assessment included the recording of a history, physical examination, evaluation of range of motion. Elbow-extension strength was measured at 45° and 90° flexion with the forearm in neutral rotation. The unaffected arm was used as a control for each patient.

#### Follow-up:

All patients were examined at monthly intervals for three months and then at six and 12 months after surgery. Each patient completed a comprehensive written questionnaire regarding functional capabilities, residual symptoms and existing disabilities. The elbow range of motion and strength were noted. Triceps strength was graded according to the system given by Wolfe *et al.*^6^ [Table 1]. Radiographs were obtained in anteroposterior and lateral projection on each follow-up visit till bony union.

**Table 1:** Grading of triceps strength

Good	Able to extend against resistance with no extension lag
Fair	Able to extend against gravity with no extension lag
Poor	An extension lag
Failure	Frank triceps avulsion

## RESULTS

The mean duration of follow-up was 18±4 months (range 12 to 36 months).

### Strength:

Thirty-five patients (87.5%) had good triceps strength, five patients (12.5%) had fair strength and one patient had an extension lag of 10°. No patient had a reoperation. No patient had triceps rupture at a minimum follow-up of 12 months.

### Range of motion:

The mean flexion contracture was 5.25° (range, 0°- 20°). Mean arc of flexion-extension was 118.4° (range, 80°-130°). All patients had some loss of flexion and extension and no patient had loss of forearm rotation. Range of motion improved in the first six months.

### Stability:

There was no evidence of anteroposterior elbow instability on manual testing. Manual stressing of the elbow in the varus-valgus plane, including provocative maneuvers for valgus and posterolateral instability, did not reveal any laxity in any patient.

### Radiographic analysis:

Union was defined as the presence of bridging callus or the disappearance of the fracture line on three of four cortices seen on the anteroposterior and lateral radiographs. Intra-articular gap or step deformity was measured directly on radiographs. Average time of healing of the distal humerus fractures was 3.2±1.6 months. There was one malunion (varus angulation of 7°). No patient had nonunion. No fracture was fixed with greater than 10° of angulation in flexion, extension or rotation. No patient had a loss of reduction or fixation. Two patients needed removal of the implant in view of the subcutaneous prominence of the hardware.

### Complications:

There was one postoperative superficial infection, which resolved after treatment with oral antibiotics and dressing. There were no deep infections. The iatrogenic radial nerve palsy was observed in two patients, which resolved completely by three months. Preoperative ulnar nerve palsy resolved completely in two months in all five patients.

## DISCUSSION

Triceps-splitting, triceps-reflecting and olecranon osteotomy are the most common posterior surgical approaches to the adult elbow. Triceps-splitting or -peeling approaches have postulated a negative effect on muscle strength on the basis of the potential for weakened reattachment, direct muscle injury with resultant fibrosis and injury to intramuscular nerve branches.^7^ In the TRAP approach the dissection is in the internervous planes and hence muscle injury with resultant fibrosis and injury to intramuscular nerve branches are avoided with this approach.

It is generally thought that a posterior surgical approach provides optimal exposure of the intra-articular aspect of the distal part of the humerus and the olecranon osteotomy is the gold standard against which other approaches are compared. However, its drawbacks (delayed union or nonunion, prominent hardware and so on) have led to other avenues of dealing with the extensor mechanism.^8^ Several authors have reported various complications associated with tension band wiring of olecranon.^9^–^12^ Macko *et al*. reported elbow symptoms due to prominent K-wire in 15 cases (75%) out of their 20 cases and skin breakdown in four (20%).^9^ In a study of 88 fractures of the olecranon, Horne *et al*. reported that 66 (75%) patients required removal of the wire within one year because of pain and 7% patients had nonunion.^10^ Ring *et al*. reported a non-union rate of 30% of transverse olecranon osteotomy in surgical fixation of fractures of distal humerus.^11^ Gainor *et al.* observed that 27% of their patients necessitated removal of hardware because of symptoms related to wires and septic olecranon bursitis.^12^ Triceps-reflecting anconeus pedicle approach can avoid such problems altogether.

The median exposed articular surface for the triceps-splitting, triceps-reflecting and olecranon osteotomy approaches was 35%, 46% and 57%, respectively.^13^ Olecranon osteotomy exposed more articular surface than the triceps-splitting approach but was not significantly greater than the triceps-reflecting approach.^13^ Extreme flexion of elbow allows visualization of most of the anterior articular surface of the humerus. We had no problem even in comminuted bicondylar fractures with triceps-reflecting anconeus pedicle approach.

In our study 87.5% of patients regained normal strength by 12 months while Askew *et al.* reported loss of strength of triceps in all patients with olecranon osteotomy or triceps-splitting approach.^14^

The TRAP approach allows extensive distal humerus exposure, including the supracondylar/intercondylar region. The TRAP approach is extensile enough in treating complex humeral fractures. Both articular reconstruction and fixation can be easily managed without creating an olecranon fracture.^15^ The repair is easy and strong enough to allow a rapid rehabilitation. No significant triceps weakness and dysfunction was observed after the TRAP approach in the treatment of the intercondylar fractures of the humerus.^15^

Our study has certain shortcomings. We measured muscle strength manually and that may insinuate the potential of subjective bias; objective testing of the muscle strength would have avoided this bias. The rate of posttraumatic degenerative changes in the elbow may increase with time, negatively affecting function and hence a longer follow-up is required.

## CONCLUSION

Triceps-reflecting anconeus pedicle approach provides good visualization for fixation of intercondylar fractures of the humerus, without any untoward effect on triceps strength and postoperative rehabilitation; and one can avoid iatrogenic fracture of the olecranon and its associated complications.
